# Development and Application of the Lincoln Adherence Instrument Record for Assessing Client Adherence to Advice in Dog Behavior Consultations and Success

**DOI:** 10.3389/fvets.2018.00037

**Published:** 2018-03-06

**Authors:** Lisanna Lamb, Nadja Affenzeller, Lynn Hewison, Kevin James McPeake, Helen Zulch, Daniel S. Mills

**Affiliations:** ^1^Animal Behaviour Cognition and Welfare Group, School of Life Sciences, University of Lincoln, Lincoln, United Kingdom; ^2^Clinical Unit of Internal Medicine Small Animals, University of Veterinary Medicine Vienna, Vienna, Austria

**Keywords:** adherence, behavior, compliance, consultation, counseling, scale, veterinary behavior

## Abstract

Adherence to the advice of medical practitioners is critical to successful treatment outcomes and has been much researched in human health, but is less well studied in the veterinary and clinical animal behavior fields. Given that the management of behavior problems often requires substantial change in established client behavior, it is likely that adherence is a substantive issue affecting success. However, little is known about the relationships between relevant factors, and there is no established way of assessing these. Therefore, the aim of this study was to develop an instrument for coding factors likely to impinge on pet owner adherence to behavior advice and validate its utility through the identification of the factors appearing to relate most closely to a successful treatment outcome in a sample population from our clinic. Potential factors affecting adherence were identified from human health and animal behavior studies, and a survey instrument developed with items matched to these factors. Forty-two dog owners who had attended the University of Lincoln Animal Behavior Clinic over a 2-year period provided data used in the analysis. The assessment of treatment outcome success by clients and clinicians was correlated, but clinicians tended to overestimate success by half a point on a 5-point scale. Eleven items relating to adherence were found to correlate with client ratings of treatment success in a univariate analysis, with three of these remaining in an ordinal logistic regression model. These three related to trust in the advice given by the clinician, concern over distress caused to the pet in the longer term and the perceived recommendation of treatment measures that had failed. By further examining the relationship between all of these factors in a hierarchical cluster analysis, we were able to postulate ways in which we might be able to improve client adherence and thus treatment success. This provides a model for the application of the instrument in any veterinary behavior practice wishing to use client feedback to rationalize areas of the consultation which might be improved.

## Introduction

In clinical animal behavior, where the majority of treatment is carried out by pet owners, adherence (also referred to as “compliance”) by the owner to the recommended treatment plan is integral to a successful outcome (1). Although compliance-related issues are recognized as one of the biggest challenges within clinical behavior practice (Ballantyne and Buller (2)), there is a paucity of empirical research on this matter. By contrast, there is an established literature on patient adherence in human medicine from more than three decades of research [e.g., Ref. (3–5)], with a more recent literature in veterinary medicine, following a landmark article by the American Animal Hospital Association (AAHA) in 2003. In human medicine, “adherence to medicines” is defined as “the extent to which the patient’s action matches the agreed recommendations” (6). This definition can be adapted to clinical animal behavior, to read: “the extent to which the *owner*’*s* action matches the agreed recommendations.”

In their review of research on nonadherence in human medicine, Martin et al. (4) report an average adherence by patients to treatment recommendations of 60%, but where treatment is complex or requires lifestyle changes, this falls to 30%. Similar results were seen in the AAHA (7) study of veterinary medicine which reported an overall adherence of 64%, reducing to 20–30% for certain treatments. Adherence results specific to clinical animal behavior are not available, but in a study of pet owner adherence, Talamonti et al. (8) report that pet owners have more difficulty applying new behavioral management rules than any other form of veterinary treatment. It is therefore likely that adherence rates in clinical animal behavior management may be very low, although it is clearly integral to the actual success of behavior consultation. Only if we know about adherence to a program can we really evaluate the effectiveness of a theoretical treatment, and a theoretical treatment which cannot be implemented is of little value to a client. It is therefore essential that we understand more about the process and factors affecting client adherence in problem behavior management.

Within the human health field, over 200 different variables potentially associated with nonadherence have been studied, and there are as yet no reliable models to predict patient nonadherence (5). Nonetheless, relevant factors can be summarized into a smaller number of categories, broadly grouped into those relating to communication between the doctor and the patient, the treatment program, factors associated with the doctor and factors associated with the patient [Table 1 derived from Jin et al. (3), Martin et al. (4), and Vermeire et al. (5)]. Of these, “communication” (4, 9) and the “relationship between doctor and patient” (10–12) are reported to have the greatest effect on nonadherence. In the human–child psychology literature, it is also reported that nonadherence by a parent not only impacts directly on the opportunities given to the child to learn new behavior (13) but also the relationship between the therapist and the parent, potentially leading to the therapist wanting to spend less time to help the parent, which exacerbates the problem for the child further (14). These considerations are particularly pertinent when it comes to studying adherence in the context of pet problem behavior management by clients, where treatment often involves the primary carer following the advice of the clinician in order to bring about an effective behavior change independently. Clearly, the motivation of the primary carer to engage with treatment is key in these contexts, but even in the human field, it is noted that there is a lack of research into this (15).

There are also many factors associated with the treatment itself which can influence adherence in the human medical context which may be of relevance when it comes to considering veterinary behavior management. This includes the complexity of treatment (3) and its duration, with longer treatment periods negatively associated with poorer adherence (16). Lifestyle changes are also more difficult to adhere to than dosing regimes [e.g., see Ref. (9)] with individualized patient training and regular follow-up recommended in the former circumstances to maximize adherence. Patients who lead busy lives also have more difficulty finding time to implement the treatment, resulting in lower adherence (17), and, perhaps not surprisingly, when there are perceived negative side effects associated with the treatment (18) or patients are skeptical about the treatment (19, 20), adherence levels reduce.

Within the veterinary field, it has been estimated that the overall adherence rates may be around 64%, with practices overestimating this figure by as much as 50% for some conditions (7). This study has prompted a change in thinking about adherence within veterinary medicine, with a shift away from client blame. In one of the few studies to consider the issue within the veterinary behavior context, Mills and White (21) found that poor adherence with simple procedures, which owners had previously followed and found successful, appeared to be the main reason for the apparent decline in the long-term efficacy of the treatment of feline urine spraying. Blackwell et al. (22) also found that although providing written advice to owners of the dogs newly rehomed from a shelter could reduce the risk of separation-related problems, poor adherence to the advice, especially elements requiring significant effort or lifestyle changes, was a major barrier to the success of the program. Jobling and Creighton (23) have also reported that in the case of horse clients, adherence to advice is better if the benefits are demonstrated early in the consultation process and if there is an emphasis on external causes which can be controlled. Casey and Bradshaw (1) provided a useful description of factors likely to influence owner adherence and the success of treatment of behavior, many of which reflect those identified in the human medical literature. The level of adherence was found to relate to the type of presenting problem, to the relative complexity of the treatment program and the client’s perception of the clinician. However, none of these studies have examined the reasons why clients did not adhere to the advice given.

One of the greatest challenges in conducting adherence studies lies in the measurement of adherence. In human medicine, the question of “how to measure adherence” has prevented the development of a standard method of measurement (5) and has been a barrier to comparing different studies. Measures used include pill counts, self-reports, electronic measures, and biological markers (4), with a multi-method approach being frequently advocated, but some of these measures are not feasible in contexts such as animal behavioral therapies (1) where treatment is administered by owners usually at arm’s length from the clinician. Self-reported measures of adherence are often inaccurate with patients often exaggerating adherence levels (24), whereas clinician reports may be a more acceptable method of measuring adherence (25).

There is clearly a need for further work aimed at identifying the factors related to nonadherence, especially within the field of clinical animal behavior consultations, where it is widely recognized that the consultation process is integral to the successful implementation of the advice given (Paris, 2002). While specifying factors of nonadherence at higher levels, such as clinician communication, can be useful for raising awareness of the general issue of adherence, it is essential that factors are identified and described as specifically and objectively as possible in order to identify the precise issue so that it can be addressed efficiently. Therefore, the aims of the present study were first to identify and organize items relating to communication between the clinician and the client, as well as the treatment program, which might be associated with adherence to clinical animal behavior advice in order to produce an instrument for assessing client adherence in a clinical context [The Lincoln Adherence Instrument Record (LAIR)]. We specifically excluded personal factors associated with the clinician and the client at this stage in order to focus on the generic skills of consultation. Our second aim was to illustrate the use of LAIR using our own clinical dataset in order to examine which factors were associated with treatment success at the University of Lincoln Animal Behavior Clinic.

## Materials and Methods

### Survey Development

Topics of interest were identified from a review of factors associated with nonadherence in human health (Table 1) and those described in relation to clinical animal behavior by Casey and Bradshaw (1). These could be broadly classified into three levels (Table 2). The top level category (Primary/Level 1) described the broad issue, i.e., practical barriers to success, client understanding, client confidence, and support networks. The next level (Secondary) considered general functional issues, such as “time,” “distress caused by the recommendations,” etc., while the final tertiary level described specific issues, e.g., “Treatment took too long to administer,” “Owner’s life considered too busy,” etc. These were then used to generate statements for the client interview/survey. For example, the nonadherence item “Treatment took too long to administer” was assessed using a rating of the statement: “Implementing the treatment did not take up too much of my time each day.” Reponses were assessed using a 5-point Likert scale from “strongly disagree,” through “disagree,” “neither disagree nor agree,” and “agree” to “strongly agree”. Items were structured so that there was a good balance between agreement and disagreement indicating nonadherence. Items could be further classified according to whether they related to the consultation, report, or implementation of the treatment (Table 2). In the survey, clients were also asked to assess the extent to which they agreed with the statement that their pet had been successfully treated (see Appendix S1 in Supplementary Material for a full copy of the survey instrument). The survey was piloted with six volunteer clients to ensure clarity and comprehensibility and to test the collection of data *via* the online survey system, Survey Monkey™. Behavior clinicians were also asked independently to review each case and score their agreement with the statement relating only to whether the pet was successfully treated (success score), in the same way as clients (see Appendix S2 in Supplementary Material).

**Table 1:** Factors associated with nonadherence in human medicine from Jin et al. (3), Martin et al. (4), and Vermeire et al. (5).

Cause category	Cause of nonadherence
Communication	Poor communication
	Lack of patient involvement in decision making
	Duration and frequency of interaction with doctor
	Ignorance of nature of the disease and treatments

Treatment Program	Complexity of treatment
	Degree of behavioral change required
	Physical difficulties
	Treatment duration
	Time constraints
	Treatment cost
	Number of medications
	Features of a disease
	Clinical setting
	Side effects

Doctor	Doctor’s attitude and empathy toward the patient
	Doctor–patient relationship
	Doctor’s interpersonal skills

Patient	Patient attitudes, beliefs, and group norms
	Cultural variations
	Patient’s beliefs about medicine
	Patient cognitive ability
	Psychiatric disorders
	Demographic variables (age, sex, social class, etc.)

**Table 2:** Organization of factors associated with nonadherence to advice given at behavior consultation including related Lincoln Adherence Instrument Record survey items.

Broad issue	Functional issue	Specific issue	Survey item	Item no.
Practical barriers to implementation	Time	Treatment took too long to administer	Implementing the treatment did not take up too much of my time each day.	13
		Owner’s life considered too busy	I found treatment easy to fit into my busy life.	14
	Distress	Immediate distress to pet	Implementing the treatment plan caused my pet immediate distress.	17
		Longer-term distress to pet	Implementing the treatment plan resulted in my pet being distressed over time.	18
		Longer-term behavioral issue	Implementing the treatment plan caused other behavior problems in my pet.	19
		Owner distress	Implementing the treatment plan caused me distress	15
	Physical resources	Financial cost	Implementing treatment plan was too expensive	16
		Too physically demanding for owner	I found the treatment plan physically demanding	25
		Too physically demanding for pet	The treatment plan was too physically demanding for my pet	26
	Change	Changes to daily routine	Implementing the treatment plan caused significant changes to my daily routine	23
		Changes to lifestyle	Implementing the treatment plan caused significant changes to my lifestyle	24

Client understanding	Consultation-related factors	Terminology used by clinician	The clinician used terminology I did not understand	1
		Insufficient explanation of advice	I didn’t understand the advice given in the consultation	2
		Uncomfortable environment	The consultation took place in a comfortable environment	3
		Occurrence of distractions	I became distracted during the consultation	4
		Consultation too long	The consultation was too long	5
		Consultation too short	The consultation was too short	6
	Report-related factors	Complex advice	The treatment plan was too complex	8
		Too much information	The clinician’s report contained too much information	9
		Too little information	The clinician’s report contained too little information	10
		Technical terminology	I understood all the terminology used on the treatment plan	11

Client confidence	Belief in recommendation	Trust in the advice of the clinician	I trusted the advice of the clinician	7
		Previous negative experience of the intervention	I had tried the recommended advice previously and it did not work	12
	Treatment failure	Failure to implement all advice	I followed all advice recommended in the treatment plan	20
		Failure to implement advice correctly	I followed all advice recommended in the treatment plan to an appropriate standard	21
		Failure to implement advice for long enough	I followed the treatment plan for the recommended amount of time	22

Support networks	Clinic support	Not asking for support from the clinic	I asked for support from the clinician when I needed it	27
		Lack of response from the clinic	The clinician provided support whenever I asked	28
	Personal support	Lack of support from friends and family	I had sufficient support from friends and family to implement the treatment plan.	29

*Light gray items relate to the consultation, dark gray items to the report, and white items the implementation of treatment*.

### Participants

Participants for the survey were drawn from a convenience sample of willing clients who had given permission for their further involvement in our work and who had attended a behavior consultation at the University of Lincoln Animal Behavior Clinic with a dog in the 2-year period from July 2014 to May 2016, covering a broad spectrum of treatment success outcomes. A 2-year time frame was used to provide a sufficient number of participants within a time frame in which they are likely to have good memory recall. From an initial population of 86 clients selected, three were excluded because the initial outcome was euthanasia or rehoming, a further five were not eligible (e.g., no client permission for the case to be used in teaching and research).

Participants were sent an initial email explaining the purpose of the study and that they were being contacted by a member of the team who was not involved in the consultation and treatment process. Furthermore, owners were assured that their responses would be anonymized so that the clinicians involved would not be able to link their response to the case within the dataset, together with a link to the survey website. After 2 weeks, participants who had not completed the survey were emailed again. After a further 3 weeks, participants who had not completed the survey were called to ask if they would take part.

Seventy-eight clients were contacted by email and telephone to request their participation in the study, 54 provided data but 12 did not give client identity for use in the study, leaving 42 identifiable respondents in the final analysis for which both full client and clinician data were available.

### Data Analysis

All statistical analyses were conducted using Minitab 17. Data relating to ratings are of an ordinal nature, and normality tests revealed that they were not normally distributed; therefore, non-parametric tests were used throughout. Descriptive summary statistics were calculated for both client and clinician success rating, and the relationship between the two was assessed to establish the quality of the dataset, i.e., that there was a good coverage of treatment outcomes and that these were reliable.

In order to describe the mathematical relationship between items, a hierarchical cluster analysis using Ward’s method of linkage was undertaken. As the items making up the model consisted of factors expressed in both a positive and a negative way, those that were expressed positively were reverse-scored to align with the potential issue with adherence that they reflected. This was then examined to identify whether items grouped in accordance with the rational organization undertaken at a primary, secondary, and tertiary level, or whether there were other associations that could be rationalized and provide insight into the relationship between different elements of the consultation process.

In order to examine which factors were associated with treatment success, we initially undertook a univariate analysis using Spearman rank correlation coefficients between each of the 29 items and owner ratings of treatment success. Factors that were significant at a level of *p* < 0.2 were then taken forward into an ordinal logistic regression model (26) using a logit function, with nonsignificant factors eliminated serially on an individual basis according to their significance in order to generate a final model with only significant factors.

## Results

Owners, on average, disagreed or disagreed strongly with each of the specific items being an issue (median scores 1, 1.5, or 2 for all item scores, Table 3). For nine items, owner responses covered the full range of scores possible, and for a further 11 items, they scored from strong disagreement to agreement with the item being an issue. All clients disagreed or disagreed strongly that the terminology used by the clinician, the level of explanation given by the clinician, or the consultation being too long were issues.

**Table 3:** Summary of client ratings (minimum, maximum, and median scores) of specific issues associated with nonadherence from behavior consultation and their significance with respect to treatment success.

Item no.	Specific issue	Min	Max	Median
1*	Terminology used by clinician	1	2	1
2	Insufficient explanation of advice	1	2	1
3	Uncomfortable environment	1	3	1.5
4	Occurrence of distractions	1	4	1.5
5	Consultation too long	1	2	1
6	Consultation too short	1	3	1
7*	**Trust in the advice of the clinician**	**1**	**5**	**1**
8*	Complex advice	1	4	2
9*	Too much information	1	3	1
10*	Too little information	1	3	2
11*	Technical terminology	1	5	1.5
12*	**Previous negative experience of intervention**	**1**	**5**	**2**
13	Treatment took too long to administer	1	4	2
14	Owner’s life considered too busy	1	4	2
15	Owner distress	1	4	1.5
16	Financial cost	1	3	2
17*	Immediate distress to pet	1	5	2
18*	**Longer-term distress to pet**	**1**	**5**	**1**
19	Longer-term behavioral issue	1	4	2
20	Failure to implement advice	1	5	2
21*	Failure to implement advice correctly	1	4	2
22	Failure to implement advice for long enough	1	4	2
23	Changes to daily routine	1	5	2
24	Changes to lifestyle	1	5	2
25	Too physically demanding for owner	1	5	2
26	Too physically demanding for pet	1	4	1
27	Not asking for support from the clinic	1	4	2
28	Lack of response from the clinic	1	3	2
29*	Lack of support from friends and family	1	4	2

Item number refers to the original survey, whereas the specific issue relates to the expression of the item in such a way that a high score (max 5) indicates that the client agrees strongly with this being an issue, whereas a low score (minimum 1) indicates that they disagree strongly with this being an issue. Items have been converted into issues, as some original items were expressed positively and some negatively. Ratings on items with “*” were negatively correlated with treatment success in a simple univariate analysis, and items in bold remained significant in the final logistic regression model.

Response ratings by owners to the item asking “Was the pet successfully treated?” ranged from “Strongly disagree” (score 1) to “Strongly agree” (score 5) with an average rating within the sample of “Neither agree nor disagree” (median score 3, mean 3.4). The equivalent data from clinicians had a range from “Neither agree nor disagree” (score 3) to “Strongly agree” (score 5) with an average rating of “Agree” (median score 4, mean score 3.81), indicating good coverage. There was a significant correlation between the owner and the clinician ratings (rho = 0.383, *p* = 0.012), but also a significant difference between the two (*W* = 106.5, *p* = 0.017, median difference = 0.5), with clinicians tending to rate score more favorably by half a rating. Given that problem behavior is a subjective construct of the client (27) and that their responses covered the full range of outcome ratings, owner ratings of success were used as the dependent variable to be predicted from the ratings of their perception of various elements of the consultation process.

**Figure 1:**
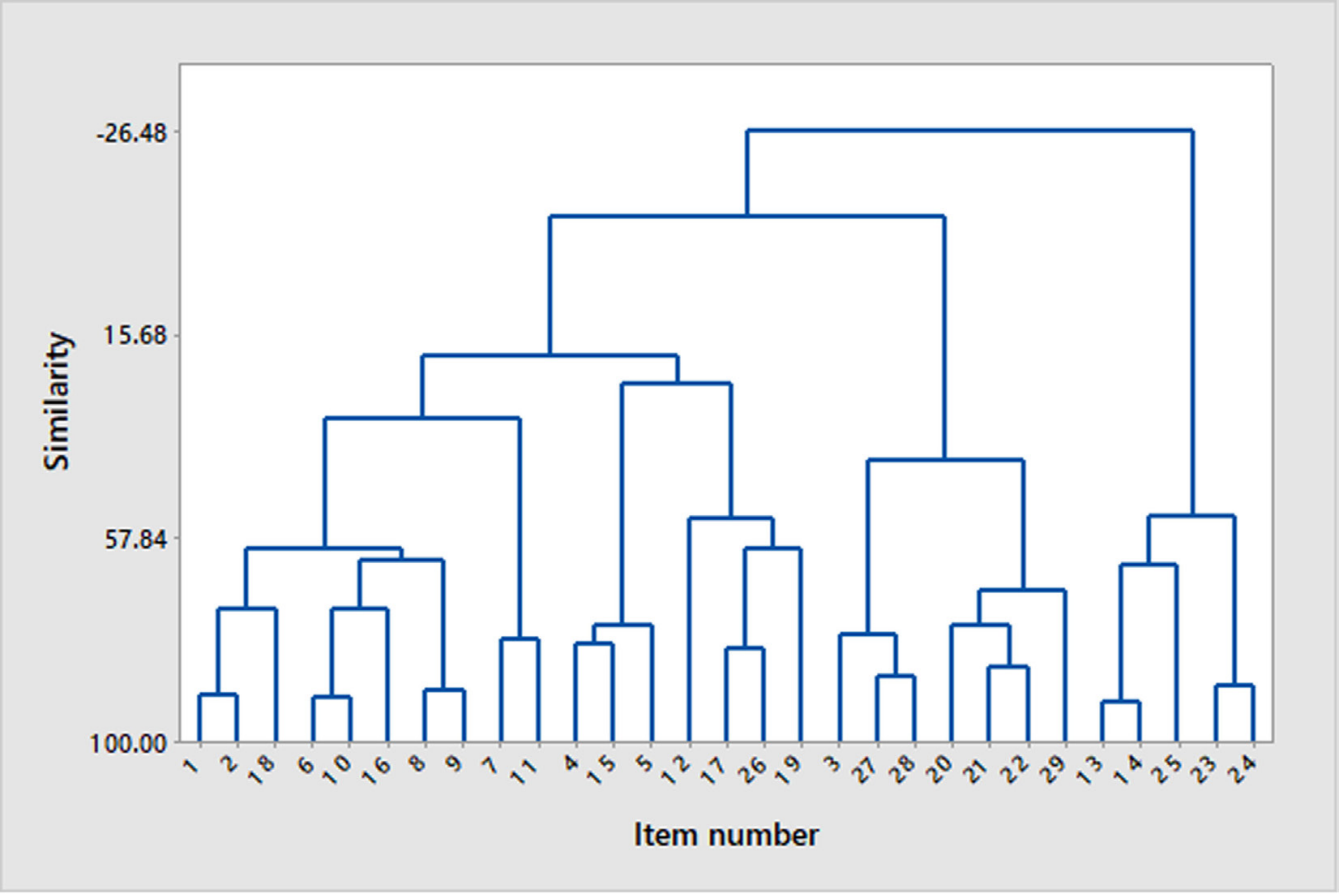
Dendrogram showing clustering of survey items on the basis of similarity in response score using Ward’s method of linkage. For details of item content, see Tables 2 and 3.

The hierarchical cluster analysis of these perceptions of the consultation and treatment plan revealed four major clusters of items (Figure 1). We describe these in the following text together with the median score provided by clients, where a score of 5 indicates the most favorable response and 1 the least favorable response (since some items were expressed in a way where agreement indicated an issue and others in a way where agreement meant there was no issue) The most distinct cluster (which for convenience, we refer to as the cluster relating to the *integration of treatment by client*) consisted of the items: time required to implement the program (item no. 13), how easy the program was (14), its physical demands (25), and changes to the owner’s routine (23) or lifestyle (24). The next cluster (which we refer to as *client focus on success*) consisted of the items relating to the comfort of the environment in which the consultation took place (3), the need (27), and availability (28) of support from the clinician together with those relating to the actual ability to implement the advice given (20), the quality of implementation (21), and the duration of implementation (22) alongside the availability of supportive social networks (29). The third cluster (which we refer to as *client doubt*) was a largely looser series of connections between the items relating to the distraction of the client during the consultation (4), treatment implementation causing the owner distress (15), the consultation being too long (5), the recommendation of advice tried previously by the client (12), the risk of immediate distress being caused to the pet by the advice given (17), the treatment plan being too physically demanding for the pet (26), and causing other behavior problems (19). The final cluster (which we refer to as *client appreciation*) contained items relating to the use of terminology that the client did not understand (1) or wider misunderstanding of the advice (2), the implementation of advice causing distress to the pet over time (18), the consultation being too short (6) and having too little information (10), treatment being perceived as too expensive (16), too complex (8), and the written report containing too much information (9), alongside the trust the client had in the clinician’s advice (7) and the client’s understanding of terminology in the treatment plan (11).

The ratings of 11 items expressed in terms of the potential problem they might pose to success correlated significantly (and negatively) with client ratings of treatment success in the univariate analysis. These were the items relating to the use of terminology that the client did not understand (item no. 1) (rho = −0.325, *p* = 0.026), lack of trust in the advice given by the clinician (7) (rho = −0.552, *p* < 0.001), the complexity of the treatment plan (8) (rho = −0.476, *p* = 0.001), too much information in the clinician’s report (9) (rho = −0.452, *p* = 0.001), too little information in the clinician’s report (10) (rho = −0.326, *p* = 0.025), lack of understanding of terminology used (11) (rho = −0.319, *p* = 0.029), the inclusion of treatment recommendations tried before (12) (rho = −0.408, *p* = 0.004), implementation causing immediate distress to the pet (17) (rho = −0.325, *p* = 0.026), implementation causing distress to the animal over time (18) (rho = −0.532, *p* < 0.001), not following all the advice to an appropriate standard (21) (rho = −0.353, *p* = 0.015), and lack of support from friends and family to implement the treatment plan (29) (rho = −0.299, *p* = 0.041).

Assumptions concerning the goodness of fit and gradient of slopes not being zero within the final ordinal logistic regression model were met. This model contained three items: lack of trust in the advice given by the clinician (odds ratio and 95% confidence interval: 2.09, 1.02–4.31), the inclusion of treatment recommendations tried before (2.64, 1.39–5.02), and implementation causing distress to the animal over time (8.05, 2.39–27.11). Measures of association between the response variable and predicted probabilities indicated a generally good predictive performance by the model with 78.7% concordance, 15.5% discordance, and 5.8% ties.

## Discussion

Through the creation of LAIR, this is the first study, to the authors’ knowledge, to systematically identify and examine the impact of factors believed to underpin client adherence to behavioral advice on treatment outcome. Although the results relate to our own practice and cannot be generalized, the production of the instrument provides a means to assess which of the factors identified in the literature as being important to client adherence are relevant to any given clinic or a clinician. Previous studies on owner adherence in the field have not allowed this level of specification, being based on much more general measures such as composite scores [e.g., Ref. (1)], or examining particular elements within a program (28) or using a small number of specific indicators [e.g., Ref. (8, 29)] to explore how these relate to the changes reported over time. Other studies have examined specific aspects of adherence indirectly, for example, by examining the relationship between the number of pieces of advice given and treatment success (30) or the interaction between different elements such as the use of psychoactive medication and compliance with behavioral modification exercises (29). This is also believed to be the first study of follow-up outcomes in the field of veterinary behavioral medicine to take specific measures to protect the integrity of the information obtained from clients by using an individual not connected with the consultations and treatment process to undertake the survey. Providing other assurances to clients concerning specific case outcome anonymity (blinding of clinicians as to the client rating of their consultation) may be critical to gaining their confidence and an honest evaluation. As a result, we were able to capture data from cases covering the full spectrum of owner-rated success (scores 1–5). With respect to the individual items, for 21/29 (72.4%) of them, client responses covered at least 80% of the 5-point scale of items (score ranges from 1 to at least 4). Taken together, this indicates excellent variability within the data for modeling the relationship between items.

The pivotal AAHA (7) publication on adherence in veterinary work reported that while most practices believed that a high percentage of their clients were adhering to recommendations, a much smaller percentage of clients were actually adherent, suggesting that clinicians can overestimate adherence. In our study, clinicians were not asked about adherence to these factors, but rather they were required to assess the outcome, and our data indicate that they tend to overestimate this too. The clinicians never clearly disagreed with the treatment being a success and the clinician’s assessment was typically half a point higher on the rating scale. Nonetheless, the ratings of clients and clinicians did correlate. This may reflect a resistance by clinicians to accepting failure or a more optimistic (and possibly more realistic) assessment of what can be reasonably achieved in the circumstances.

While our initial classification of issues affecting adherence grouped different elements of the actual consultation, report, and implementation into functionally meaningful categories at several levels (Table 2), it was not expected that this would reflect the grouping of the specific issues in practice. Nonetheless, it is worth noting that in our own clinic’s case analysis, no items relating to issues at the secondary levels of time (13 and 14), physical resources (16, 25, and 26), change (23 and 24), or clinic support (27 and 28) correlated with treatment success, indicating that our general performance in these was not predictive of success. Likewise, of the four clusters identified, none of the issues relating to the cluster associated with the integration of treatment into the owner’s life were predictive of response outcome. Only two out of the seven items included in the cluster relating to the client’s focus on success were predictive of treatment outcome: failure to implement advice to an appropriate standard and lack of support from friends and family. The latter finding is consistent with the findings in human medicine, where social support is considered to be an important aid to adherence (31). It is also worth noting that it was the quality of treatment implementation and not its duration or even all elements that were predictive of outcome. Thus, these results would seem to suggest that it may be particularly important for clinicians to ensure that clients have supportive networks that ensure appropriate implementation of their advice, rather than emphasizing the duration or necessity to cover the full range of treatments recommended. In other words, high-quality-specific advice may be best, although it should be acknowledged that neither of the two items within this cluster remained in the final multivariate model.

Within the cluster referred to as client doubt, two out of the seven factors making up this cluster significantly correlated with response outcome (treatment causing immediate distress to the pet and previous negative experience with the intervention), with previous negative experience of an intervention remaining significant in the final multivariate model. From the dendrogram (Figure 1), it is clear that this latter factor joins with three others, including the perception of immediate distress being caused to the pet, the creation of other behavior problems, and the plan being too physically demanding for the pet. This would seem to suggest that these are the major concerns associated with previous failed treatments and that clinicians need to make a special effort to address these perceptions if they want clients to adopt variants of treatments which have previously failed.

Finally, within the cluster relating to client appreciation, 7 out of the 10 items were correlated with treatment outcome (Table 3); only client understanding of the advice in the consultation, the consultation being too short, and the cost of treatment were not significant in the univariate analysis. It is worth noting that all clients at least disagreed that there was a problem with understanding the clinician during the consultation (which would tend to indicate that this aspect of our consultations is being executed very effectively) and that there was little variation in this (Table 3); this issue may be a potentially important factor in other situations. Indeed, the closely related item referring to the technical nature of the terminology used did correlate at a univariate level with treatment outcome. This suggests perhaps that small amounts of misunderstanding might have a big impact on treatment outcome. In support of this, another significant grouping at the univariate level includes the client’s perception that the treatment plan was too complex and that there was either too much or too little information in the report. Of these, too much information seems to be most closely related to the client perceiving it as too complex, potentially indicating that it is important to be clear and concise if clients are not to be overwhelmed by what is being asked of them. However, they must also have enough information to understand that what is being asked is in the animal’s best interest, since this too features in this cluster. Of the significant factors identified in the univariate analysis within this cluster, both treatments perceived to cause longer-term distress to the pet and trust in the advice of the clinician remained significant in the final multivariate model, with the welfare concern being particularly important given its effect in the model (odds ratio of around 8). From the dendrogram, it seems that trust in the clinician is closely linked with an understanding of treatment terminology, whereas concern over distress being caused in the longer term is most closely linked to the two aspects of client understanding associated with the actual consultation (general terminology used and understanding of advice). Together, these two findings emphasize the importance of clients understanding the problem and rationale for treatment in order for them to appreciate the value of advice being given and thus adhere to it, especially in the face of concerns about the distress of their pet in the longer term. It is worth emphasizing at this point that our clinic’s strategy is to focus on providing solutions to problem behavior that support animal welfare, except where the risks outweigh this, e.g., the use of muzzle restraint to prevent the risk of others being bitten. Nonetheless, even a measure like this is introduced with a focus on positive reinforcement, but given the findings of this study, it would seem that perhaps we need to more clearly address clients’ concerns about the welfare of their pet in whatever context they arise, using a language they can understand. It is unsurprising that an owner will be reluctant to follow the treatment program which they perceive as causing distress to their pet, even if it is for its longer-term benefit. Parallels can be drawn with the side effects associated with human medicine; Jin et al. (3) found that in all the studies they reviewed, side effects threatened patient adherence, and DiBonaventura et al. (18) have concluded that side effects are significantly associated with nonadherence to treatments for schizophrenia, despite the impact of this condition on the patient’s ability to function.

Overall, it is clear that among our clinic’s clientele at least, concern over distress being caused to the pet in the long term is a very potent factor related to treatment outcome and probably adherence to the advice given as a result. This might be a feature specific to the demographic of our clinic, but this nonetheless emphasizes the importance of providing compassionate support focused on the welfare of the pet and in a way that the owner can appreciate. However, it should be noted that while clients clearly care about their pet’s welfare, the way they care for their pet (i.e., what they do practically for it) may not always reflect these high levels of concern for its well-being. This might possibly be due to a misunderstanding of the animal’s needs and how it perceives its environment, with a disparity between what the animal needs and what the client thinks it needs or wants at the heart of the development and maintenance of some behavior problems. In this situation, the clinician must educate the client using a simple language and techniques that they can understand and believe in. Given the current results, it can be postulated that such cases may be particularly challenging cases to deal with, and further work could investigate the relationship between clients’ understanding and conceptualization of animal welfare with treatment success. Another important area for future investigation concerns the relationship, suggested by these results, between client understanding of the treatment plan and their trust in the clinician. It seems reasonable to suggest that if clients are not convinced of the treatment’s effectiveness, they may not fully implement the recommended measures especially where these take time and effort (32). This type of relationship has been reported in studies of patients with irritable bowel syndrome in which patient skepticism about the efficacy of treatment predicted adherence levels to the prescribed program (20). Since animal behavior treatments usually require significant effort on the part of the owner and need to be applied over a lengthy period of time, if an owner is not fully convinced that the treatment will lead to a successful outcome, it is less likely that they will be willing and able to sustain a commitment to the treatment program. To counter this, we suggest that the clinician should take time to explain the merits of the treatment measures to the owner and how these can lead to the required behavioral improvement.

**Figure 2:**
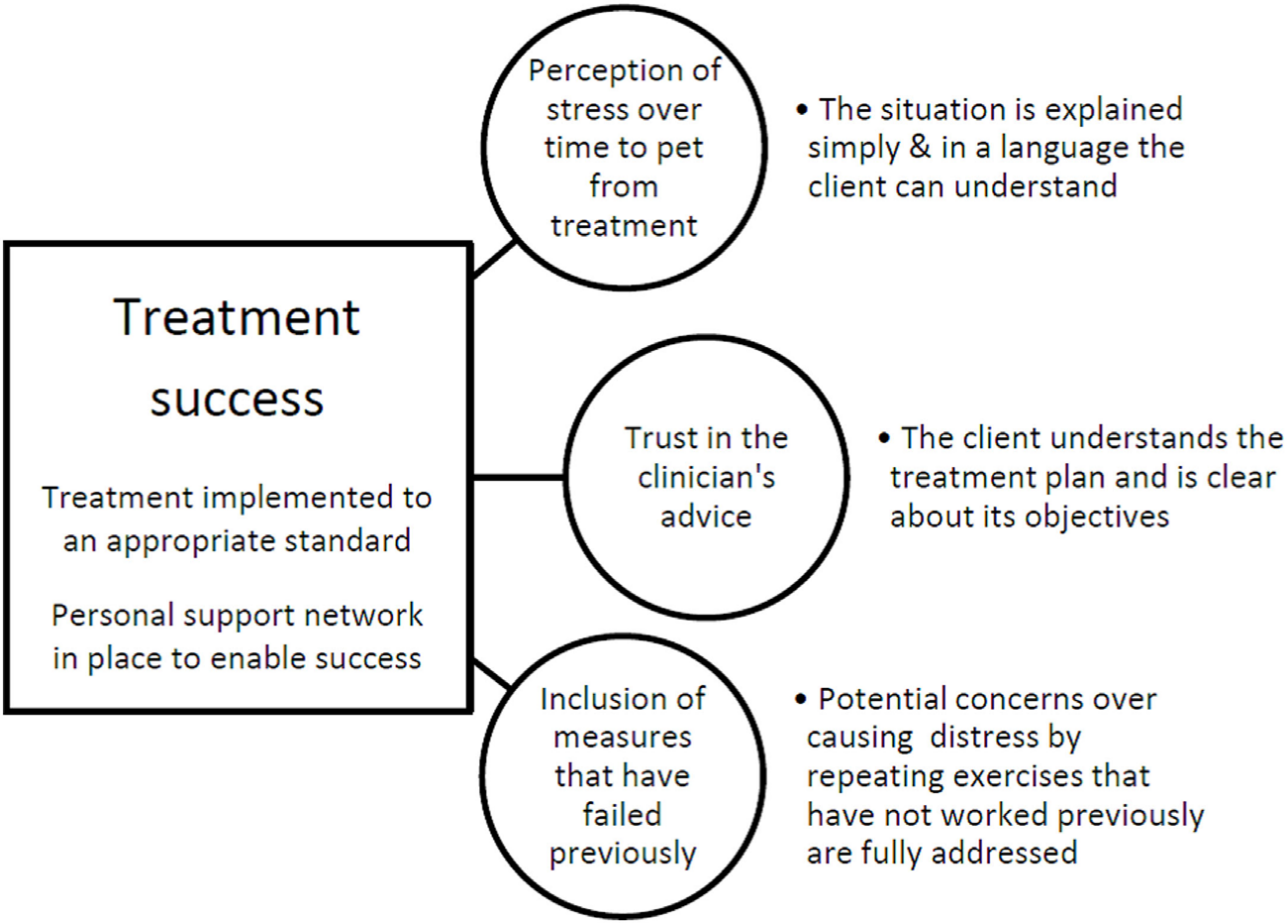
A graphical representation of postulated relationships within the caseload used in this study, illustrating points of variability associated with success. Treatment success (apparently defined by the appropriate implementation of the recommended treatment and the presence of personal support networks enabling the implementation of the treatment plan) is predicted by three key adherence factors (circles), and bullet points refer to other adherence factors that might help to mitigate against these risks.

Given the relationships identified in this study, we can postulate how they might be related and use this to prioritize areas for attention in our clinic from the feedback being provided by our clients in this way. The hypothetical relationships identified also form the basis of further research and management. With regard to the current data, we have illustrated a proposed relationship between all of the factors found to be significant at the univariate level with the three items remaining in the multivariate analysis that predicted treatment outcome as shown in Figure 2. It is important to appreciate that although factors may have been found not to be significant predictors of success in the current caseload, this does not mean that they are not important in general or in other practices. For example, if adherence is consistently high due to good practice, there may not be sufficient variation for them to predict the variation in treatment outcome. We suggest in Figure 2 that, for out clinic, treatment success might be defined alternatively by the appropriate implementation of the recommended treatment and the presence of personal support networks to enable the implementation of the treatment plan. This is because the latter two correlated with treatment success in the univariate analysis but did not remain in the multivariate model, nor did they cluster with any of the adherence issues which did remain. The three key adherence factors that did remain in the model are described in the circles. Bullet points next to these describe the practical elements that might help to mitigate against these risks derived from an assessment of the adherence factors which tended to cluster with these three key factors.

In conclusion, this study has developed an instrument and methodology to allow clinical animal behaviorists to evaluate their performance in encouraging client adherence in relation to the way they operate their consultation, the form of their report, and the implementation of treatment. It is recommended that the survey be undertaken by an individual who was not part of the team involved in the management of the pet in order to encourage clients to provide honest information. By examining the relationship between the items in the instrument using both descriptive clustering and multivariate regression, it is possible to postulate causal relationships from which the team can learn how they might best improve their service.

## Data Availability

The data are available on request from the corresponding author.

## Ethics Statement

This study was approved under the delegated authority of the University of Lincoln, School of Life Sciences Research Ethics Committee. It was conducted as part of the MSc of LL and subject to local evaluation in combination with the terms laid out in ethical approval CoSREC281 which covers the reporting of clinical case material.

## Author Contributions

LL, HZ, DM, KM, LH, and NA designed the study. LL, HZ, KM, LH, and NA executed the study. DM and LL analyzed and interpreted the data. LL, DM, HZ, KM, LH, and NA were all involved in the writing of the paper.

## Conflict of Interest Statement

The authors declare that the research was conducted in the absence of any commercial or financial relationships that could be construed as a potential conflict of interest.
