# Supplementary material for: Development and Application of the Lincoln Adherence Instrument Record for Assessing Client Adherence to Advice in Dog Behavior Consultations and Success
Source: Front Vet Sci. 2018 Mar 6;5:37. doi: 10.3389/fvets.2018.00037 (PMC5845580; doi:10.3389/fvets.2018.00037)
Supplement: Supplementary file 1 [file Table_1.docx]

**Supplementary Material**

**Appendix 1. Client Questionnaire**

Thank you for completing this questionnaire. The data collected in this questionnaire will be used in a study on how clients perceive the behaviour consultation process. We believe that this is a very important study as in better understanding this we can structure our services more appropriately.

In this questionnaire you will be asked to answer questions relating to the consultation that you attended at the University of Lincoln Animal Behaviour Clinic. If you have attended more than one consultation please only answer questions in relation to your first meeting.

The questionnaire will typically take 10-15 minutes to complete. Please note that your participation in this study is voluntary, and your participation in the study will in no way influence the treatment advice you receive from the Animal Behaviour Clinic in the future.

By carrying out the survey you consent to the information you have given to be used in this research project. You may decide to stop being a part of the research study at any time (up until any writing up of results) without giving an explanation. You have the right to ask that any data you have supplied to that point be withdrawn/ destroyed, by contacting the University of Lincoln Animal Behaviour Clinic. You have the right to omit or refuse to answer or respond to any question that is asked of you. If you have any questions as a result of reading this information, please ask the researcher before you begin filling in the questionnaire. If you have any questions regarding the study after completing the questionnaire, please contact the researcher. The researcher can be reached on *****. If you have any questions regarding your pet’s behaviour, please contact the clinic team on 01522 835475.

The data we collect will remain confidential and will be anonymised prior to analysis.

The questionnaire will be split into four sections, questions relating to; the consultation itself, the consultation report, implementing the treatment plan and additional questions. 

When answering the questions please select the appropriate box against each of the statements based on how strongly you either disagree or agree with each statement. It would be appreciated if you could provide an answer to all questions.

The answer to the first question below is the participant number which you received in your email.

|  |  | Score |
| --- | --- | --- |
|  | **During the Consultation** |  |
| 1 | The clinician used terminology I did not understand. |  |
| 2 | I didn’t understand the advice given in the consultation. |  |
| 3 | The consultation took place in a comfortable environment. |  |
| 4 | I became distracted during the consultation |  |
| 5 | The consultation was too long. |  |
| 6 | The consultation was too short |  |
| 7 | I trusted the advice of the clinician. |  |
|  |  |  |
|  | **The Consultation Report** |  |
| 8 | The treatment plan was too complex. |  |
| 9 | The clinician’s report contained too much information. |  |
| 10 | The clinician’s report contained too little information. |  |
| 11 | I understood all terminology used in the treatment plan. |  |
|  |  |  |
|  | **Implementing the Treatment Plan** |  |
| 12 | I had tried the recommended advice previously and it did not work. |  |
| 13 | Implementing the treatment did not take up too much of my time each day. |  |
| 14 | I found the treatment easy to fit into my busy life. |  |
| 15 | Implementing the treatment plan caused me distress. |  |
| 16 | Implementing the treatment plan was too expensive. |  |
| 17 | Implementing the treatment plan caused my pet immediate distress. |  |
| 18 | Implementing the treatment plan resulted in my pet being distressed over time. |  |
| 19 | Implementing the treatment plan caused other behaviour problems in my pet. |  |
| 20 | I followed all advice recommended in the treatment plan. |  |
| 21 | I followed all advice recommended in the treatment plan to an appropriate standard. |  |
| 22 | I followed the treatment plan for the recommended period of time. |  |
| 23 | Implementing the treatment plan caused significant changes to my daily routine. |  |
| 24 | Implementing the treatment plan caused significant changes to my lifestyle. |  |
| 25 | I found the treatment plan physically demanding. |  |
| 26 | The treatment plan was too physically demanding for my pet. |  |
| 27 | I asked for support from the clinician when I needed it. |  |
| 28 | The clinician provided support whenever asked. |  |
| 29 | I had sufficient support from friends and family to implement the treatment plan. |  |
| 30 | The treatment plan was successful. |  |

Please list any other factors which could improve the consultation and treatment process?

Please list aspects of the consultation and treatment process which you thought were particularly good.

**Appendix 2. Clinician Questionnaire**

Was the pet successfully treated? Please circle which you feel is most appropriate.

Strongly Disagree Disagree Neither Agree Nor Disagree Agree Strongly Agree

If the treatment was not completely successful, why do think this was the case?
